# Metabolic control by the microbiome

**DOI:** 10.1186/s13073-022-01092-0

**Published:** 2022-07-29

**Authors:** Timothy O. Cox, Patrick Lundgren, Kirti Nath, Christoph A. Thaiss

**Affiliations:** Microbiology Department, Institute for Immunology, and Institute for Diabetes, Obesity & Metabolism, Perelman School of Medicine, University of Pennsylvania, Philadelphia, PA USA

**Keywords:** Microbiome, Metabolism, Metabolites, Nutrients

## Abstract

The interaction between the metabolic activities of the intestinal microbiome and its host forms an important part of health. The basis of this interaction is in part mediated by the release of microbially-derived metabolites that enter the circulation. These products of microbial metabolism thereby interface with the immune, metabolic, or nervous systems of the host to influence physiology. Here, we review the interactions between the metabolic activities of the microbiome and the systemic metabolism of the host. The concept that the endocrine system includes more than just the eukaryotic host component enables the rational design of exogenous interventions that shape human metabolism. An improved mechanistic understanding of the metabolic microbiome-host interaction may therefore pioneer actionable microbiota-based diagnostics or therapeutics that allow the control of host systemic metabolism via the microbiome.

## Background

In recent years, the rates of obesity have reached pandemic proportions. More than half of the US population is overweight or obese [1], and the prevalence worldwide is following a similar trend [2]. Obesity is strongly associated with several comorbidities indicative of systemic metabolic dysregulation, including fatty liver disease, type 2 diabetes (T2D), and cardiovascular disease. This pandemic of obesity and metabolic dysregulation is increasing mortality, morbidity, and health care costs of individuals and societies across the globe. An improved understanding of the factors that regulate metabolic homeostasis in health and disease is urgently required [3].

Emerging evidence suggests that the intestinal microbiome is an important factor regulating systemic metabolic homeostasis [4–6]. The intestinal microbiome refers to the collective genetic material of the microbes populating the intestine, which allows for a substantial diversification of the metabolic activities available to the colonized host. Thus, the genetic and biochemical composition of the microbiome will determine the metabolic activities occurring in the intestine, which can then impact the development and function of the metabolic, immune, and nervous systems [7, 8]. These interactions provide a potential mechanistic basis whereby the microbiome can regulate systemic metabolic homeostasis.

Early evidence suggesting the intestinal microbiome plays a role in systemic metabolism came from observational reports. For instance, long-term exposure to antibiotics, performed in humans more than 60 years ago, and routinely performed in livestock to this day, has consistently led to an increase in body fat mass [9, 10]. However, it was not until the 2000s that important studies in mice robustly described an altered microbiota upon obesity [11]. It was found that feeding the exact same diet to genetically obese *ob/ob* mice and their lean siblings resulted in large differences in their microbiome composition [11]. The *ob/ob* mice had a 50% reduction in *Bacteroidetes* and a corresponding increase in *Firmicutes*. Further, this altered gut microbiota has been reproduced independently in both obese mice and humans. Additional studies showed that the microbiome generally has an enhanced property of energy harvesting in obesity [4, 6, 12].

Building on these observations, several groups have since aimed to study the causal role of the microbiome in systemic metabolism. One early report suggesting causality found that germ-free mice, which are mice entirely lacking a microbiome from birth, have reduced body fat compared to mice with a conventional microbiome [4]. Moreover, upon colonization of adult germ-free mice with the microbiome of conventionally raised mice, there was a 60% increase in body fat, despite reduced food intake [4, 5]. Further transplantation experiments have since been performed using the microbiota from mouse or human donors into germ-free mice, with the result that the microbiota transplant from obese donors resulted in increased obesity compared with transplants from lean donors [12, 13]. Taken together, these results suggest that the microbiome has properties that modulate the energy balance of the host, which has important implications in human dietary regulation and metabolic disease.

Thus, there is growing attention for the intestinal microbiome as a mediator of environmental factors that influences systemic metabolic homeostasis and may contribute to the global trends of obesity and metabolic dysregulation pervasive in our post-industrialized civilization. As the microbiome field progresses, it will be critical to achieve a more detailed mechanistic understanding whereby the microbiome regulates organismal metabolism.

## Systemic metabolism and the microbiome

Systemic metabolism is a complex structure of individual metabolic processes across cells, tissues, and organ systems that intersect to orchestrate organism-wide metabolite flux. The future of understanding host-microbiome metabolic interactions lies in obtaining a finely resolved picture regarding which of these processes are influenced by the microbiome. Although this remains a relatively young field of research, significant progress has been made over the last 15 years since the initial observations of metabolic regulation by the microbiome. Here, we review several recent examples whereby the microbiome can mechanistically influence systemic metabolism. To structure this discussion, we will consider energy balance and metabolite balance in turn.

### Energy balance

**Fig. 1:**
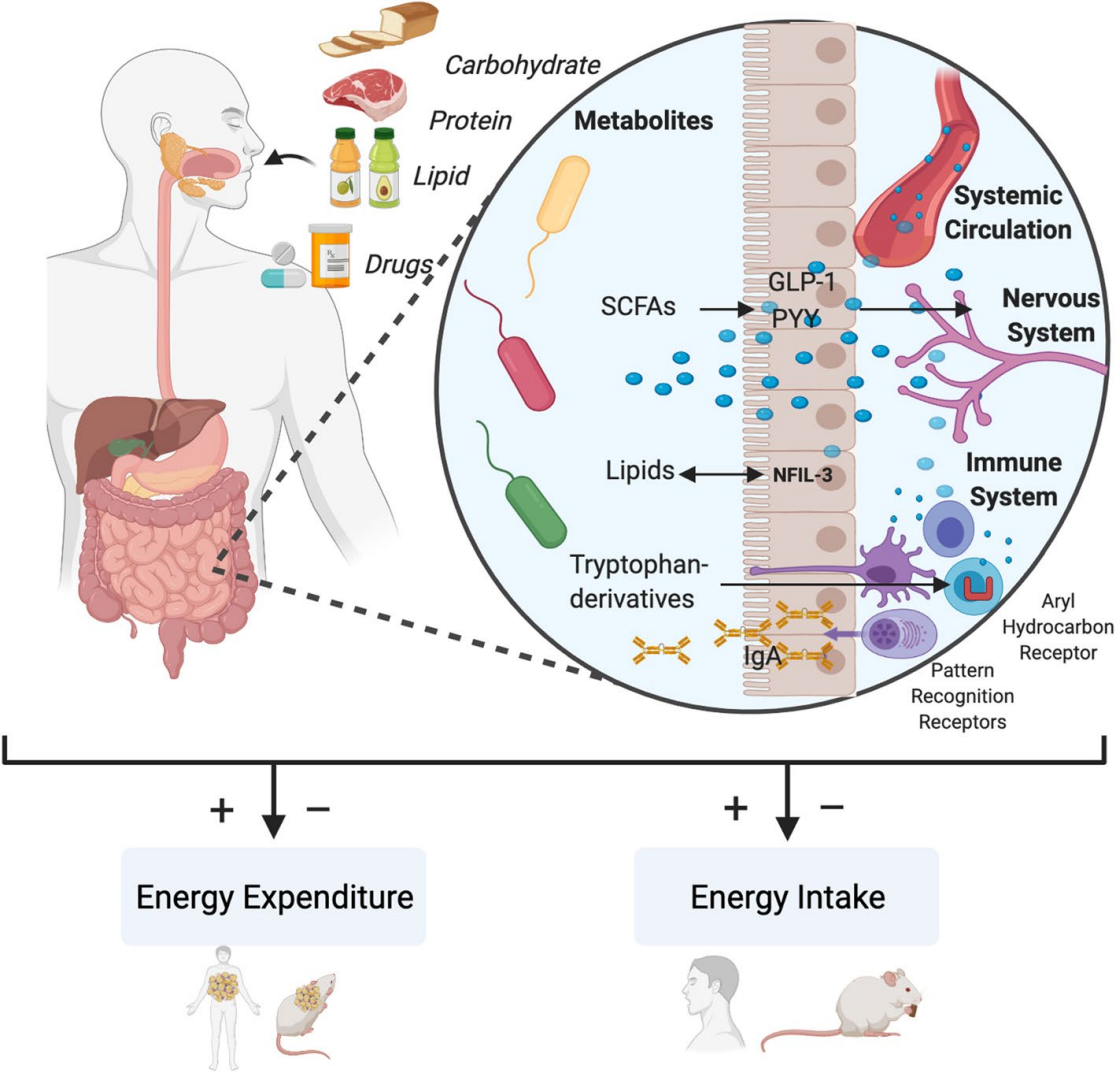
Mechanisms of metabolic host-microbiome crosstalk. Host-microbiome interaction contributes to the organismic balance between energy intake and energy expenditure. For instance, the metabolic activities of the microbiome can produce diverse metabolites such as derivatives of tryptophan metabolism, short chain fatty acids (SCFAs), and other lipid metabolites that can interact with the immune or nervous systems of the colonized host to regulate energy intake and expenditure. SCFAs induce GLP-1 and PYY release from enteroendocrine cells. Epithelial Nfil3 regulates a lipid absorption program in a microbiome-regulated manner. Tryptophan metabolites influence immune cell activities

Organisms require energy to perform the functions of life. Energy balance refers to the equilibrium between the amount of energy absorbed from food and the amount expended through the metabolic activities of the organism, the thermic effect of food, and the energy excreted in the feces or urine. The healthy human organism has a remarkable ability to adjust food intake to the need generated by the current state of energy expenditure within a very narrow window—a process which is disturbed in obesity. The role of the microbiome in these processes is still a developing field, but several recent animal studies have provided first insights into the role for the microbiome in energy balance (Fig. 1).

#### Energy intake

The microbiome can modulate energy intake directly via modulation of the digestion of complex macronutrients or indirectly via influences on hunger and satiety. Early studies in the field that connected the microbiome to energy homeostasis demonstrated that the microbiome modulates how much of the ingested energy is excreted [13, 14]. These investigations of the impact of the microbiome on obesity suggested that the microbiome of obese and lean hosts are different in composition and metabolic activity, thereby playing a role in the ability of the host to extract energy from food [13]. The mechanisms by which microbiomes differ in their overall ability to extract caloric content from food remain largely unknown, but a recent study indicated an involvement of the metabolite dimethylglycine in enhanced energy extraction and resultant weight gain [15].

In addition to the direct modulation of caloric extraction from food, the microbiome can modulate hunger and satiety signaling through interactions with the neuro-endocrine axis [16]. For example, microbiome-derived circulating short-chain fatty acids (SCFAs), products of carbohydrate fermentation by commensal bacteria, can trigger endogenous secretion of hunger-regulating peptides such as glucagon-like peptide 1 (GLP-1) and peptide YY (PYY) (Fig. 1). In particular, enteroendocrine cells of the gastrointestinal tract both express the SCFA receptors GPR (G-protein coupled receptor)-41 and GPR-43 [17–19] and secrete satiety-inducing PYY and GLP-1 once activated [20, 21]. The appetite-suppressive effects of SCFAs constitute a form of a negative feedback mechanism in response to food consumption, highlighting the role of the microbiome as a key mediator in regulating physiological adaptation to environmental factors in order to promote energy balance. Apart from SCFAs, other mechanisms might exist by which the microbiome regulates hunger and satiety. For instance, *Escherichia coli* can produce a mimic of a melanocyte-stimulating hormone (α-MSH), ClpB, stimulating PYY release and inducing proopiomelanocortin (POMC) neuron activity [22].

The relationship between the microbiome and energy intake represents an important component of metabolic homeostasis, and the microbiome has been implicated in the regulation of energy absorption and food intake, as detailed above. However, energy balance is not only a matter of energy intake, but also energy expenditure. Next, we consider the role of the microbiome from this side of the energy equation.

#### Energy expenditure

Expenditure of energy can occur by growth (anabolic) or combustion (catabolic). The role of the microbiome in growth is still a developing field and has been reviewed elsewhere [23]. Here we focus on the role of the microbiome in regulating catabolic processes that influence energy expenditure. In particular, we will focus on the process of non-shivering thermogenesis which has garnered significant interest due to its association with improved cardiometabolic health in humans [24–28].

Non-shivering thermogenesis refers to a specialized form of heat production that is facilitated by brown and beige adipocytes to maintain body temperature during cold exposure [29]. Beige adipocytes, also referred to as “brite” adipocytes, combined features of white and brown adipocytes and are induced by prolonged cold exposure in classical white adipose depots, although more efficiently in subcutaneous than visceral depots. Non-shivering thermogenesis in both brown and beige adipocytes is dependent on the protein uncoupling protein 1 (UCP-1), which dissipates the proton gradient across the inner mitochondrial membrane thereby uncoupling respiration from ATP synthesis [30]. Given the emerging interest for the role of the gut microbiome in systemic metabolism and energy expenditure, several groups have therefore studied the potential modulation of non-shivering thermogenesis by the microbiome. An early study on this topic found that germ-free mice as well as mice treated with antibiotics have improved insulin sensitivity and increased expression of Ucp1 in subcutaneous and visceral white adipose tissue [31]. On the other hand, a study published more recently found the opposite, namely that gut microbiota depletion impairs thermoregulation, reduces energy metabolism, and that recolonization of depleted microbiota partially rescues this impaired thermogenesis effect in part through the SCFA butyrate [32]. Yet another report found no effect of microbiome depletion on recruitment of thermogenic tissues or energy expenditure but did find a role for the microbiome in contributing amino acid metabolites to optimize hepatic TCA cycle fluxes in support of gluconeogenesis [33]. Thus, there is debate in the field regarding the role for the microbiome in regulating energy expenditure through non-shivering thermogenesis. Importantly, understanding the nuances of this relationship between the microbiome and non-shivering thermogenesis might provide alternative paths toward metabolic regulation for individuals in a hypercaloric or hypometabolic state [34, 35]. Whether the microbiome plays a role in regulating non-shivering thermogenesis in humans remains unchartered.

### Metabolite balance

Similar to the previously discussed mechanisms of whole-organism energy homeostasis, metabolite influx and efflux must be balanced in order to maintain health. Metabolites can function as both fuel and signals, and the same is true for microbially-derived metabolites. In the following section, we review specific metabolites and mechanisms that are regulated by the metabolic activities of the microbiome, playing a role in the metabolism of all three macronutrients: carbohydrates, proteins, and fats.

#### Carbohydrate metabolism

One of the most striking metabolic phenotypes of germ-free mice is their profoundly blunted serum glucose response to carbohydrate intake [36]. While the exact mechanisms underlying this phenomenon are not completely understood, several recent studies have contributed to our understanding. For instance, imidazole propionate was identified as a microbial metabolite produced from histidine by bacteria whose abundance is associated with T2D; imidazole propionate inhibits insulin signaling through the mTOR and AMPK pathways [37, 38], thus offering a potential explanation for the microbiome impact on systemic glucose homeostasis and providing a target for intervention.

In addition, the microbiome has been found to regulate adipose tissue biology and glucose homeostasis through the regulation of the non-coding RNA mir-181. By regulating the systemic levels of indole metabolites, which in turn suppress mir-181 expression in adipose tissue, the microbiome is involved in the transcriptional control of genes involved in insulin signaling and glucose metabolism [39].

Another area of focus is the interaction between the microbiome and enteroendocrine cells, the major glucoregulatory epithelial cells of the intestine. GLP-1 is strongly elevated in germ-free and antibiotics-treated mice, indicating an enhanced incretin effect in response to carbohydrate intake in the absence of the microbiome [40]. On the other hand, microbiome-derived metabolites, such as SCFAs and indoles, have been shown to induce GLP-1 release from enteroendocrine cells [20]. These studies complement the evidence previously described in the section on energy intake, where the microbiome can regulate hormone secretion to exert systemic influences on the host.

Recently, the microbiome has been implicated in the neuronal control of glucose homeostasis. For example, microbially-produced acetate has been found to activate the parasympathetic nervous system, thereby promoting glucose-stimulated insulin release from the pancreas in rats [41]. Propionate induces sympathetic activation, glucagon release, and insulin resistance in mice; this effect was also seen in a double-blind, placebo-controlled human study, and plasma propionate decreased with weight loss [42]. These two studies demonstrate how the microbiome can influence both nervous and endocrine elements together to tune blood sugar levels with opposing mechanism. Additionally, a microbiome-induced CART-expressing subset of neurons in the enteric nervous system can regulate blood glucose and connect polysynaptically to the liver and pancreas [43], representing interconnected neuro-endocrine control of systemic metabolism with input from the microbiome. Taken together, these studies support the notion that the microbiome has evolved mechanisms to control blood glucose via diverse autonomic and somatic neuroendocrine mechanisms. Therapeutically, each of these avenues represents a potential point of intervention that could be exogenously modulated.

#### Amino acids

Essential amino acids must be provided by the diet, and the microbiome has a major impact on the metabolism of dietary amino acids, with important effects on host physiology. For instance, the microbiome metabolizes dietary tryptophan and its metabolites, such indoles and tryptamine, which can then be sensed by the aryl hydrocarbon receptor (AHR) [44–46]. This receptor and transcriptional regulator can subsequently influence downstream physiological transcriptional programs [47]. Interestingly, some SCFAs also interact with AHR [48], suggesting a potential mechanism of cross-talk between microbiome processing of different macronutrients. Indole can also reduce intestinal permeability and stimulate the release of GLP-1 [49, 50], relating back to other GLP-1 control mechanisms discussed in the previous section.

Tryptamine and kynurenine, two potentially microbially-produced tryptophan metabolites, also modulate metabolic functions. Tryptamine can induce the release of serotonin to stimulate gastrointestinal motility [51, 52], while kynurenine has been shown to have various inflammatory, metabolic, and neurological effects [53]. Phenylalanine and tyrosine, the two other aromatic amino acids, can also be metabolized to biologically active products that modulate intestinal permeability and systemic immunity [54, 55]. Beyond the role of amino acids in metabolism and immunity, there have been reports of amino acids regulating feeding behavior in a microbiome-dependent manner. For example, in flies, the lack of any one essential amino acid from the diet produces a strong and specific appetite for proteinaceous or amino acid–rich food [56]. Remarkably, however, it was found that flies with a specific microbiome composition do not develop this protein appetite, since *Acetobacter pomorum* and *Lactobacilli* were found to be able to suppress this protein appetite. This raises the question whether in addition to local gastrointestinal metabolism, the microbiome contributes to the stability of organismal metabolites levels via a feedback loop that regulates food choice. Taken together, these studies suggest that amino acid metabolism by the microbiome has far-reaching effects, altering metabolic homeostasis, inflammation, and neurological functions.

#### Lipids

Together with carbohydrates and protein, lipids are the third major macronutrient, and their metabolism is also partially under control of the microbiome [57, 58]. As briefly described above, germ-free mice are largely resistant to high fat diet-induced obesity, excrete more lipids in feces, and have altered cholesterol metabolism [36]. The microbiome helps respond to dietary lipid changes by modulating intestinal epithelial digestive and absorptive processes in mice and zebrafish [59, 60]. Similarly, antibiotics reduce lipid absorption in rats [61]. More recent studies have focused on specific cellular actors, showing that metabolites from different bacterial species can regulate enterocyte lipid metabolism [62]. Lactate produced by *L. paracasei* promotes lipid storage in enterocytes by generating malonyl-CoA, and that acetate produced by *E. coli* promotes lipid oxidation in enterocytes by upregulating the AMPK/PGC-1α/PPARα pathway. Another study found that a previously uncultured bacterium in humans is able to convert cholesterol to a poorly absorbed sterol and is correlated with lower cholesterol levels [63]. These studies highlight the potential for developing specific bacteria- and metabolite-based therapeutic interventions to improve outcomes in for example obesity and atherosclerosis. Furthermore, dietary fat consisting of saturated fats, but not fats like fish oil, increases inflammation in WAT in a microbiome-dependent manner [64], demonstrating a link between diet, the microbiome, and systemic processes. In terms of fat metabolism, bile acids, necessary for the absorption of dietary fat, are modified by the microbiome, which transform them from primary to secondary bile acids. Bile acids themselves act as signaling molecules to control their own production and other metabolic functions [65, 66]. Widely conserved microbial bile salt hydrolases (BSH) add additional complexity to the bile acid pool [67]. BSH expression decreases weight gain, cholesterol, and triglycerides while inhibition increases weight gain [68, 69]. The interaction between bile and the microbiome represents a distinct mechanism of lipid regulation by the microbiome to impact energy intake in terms of a specific macronutrient.

A number of recent studies have added interesting facets to this general model of lipid metabolism, whereby the intestinal microbiome can interface with the immune system to influence lipid absorption in the gut. In the absence of regulation by CD4^+^ T cells, microbiome-controlled type 3 innate lymphoid cells (ILC3s) shape lipid metabolism by the secretion of IL-22, which modulates the expression of epithelial lipid transporters [70]. The same IL-22-dependent pathway of intestinal ILC3s responding to the microbiota involves the circadian clock protein nuclear factor, interleukin-3-regulated (NFIL3), which in turn regulates lipid absorption in intestinal epithelial cells [71, 72]. IL-22 production in intestinal ILC3s is also partly dependent on free fatty acid receptor 2 (FFAR2), which is agonized by microbially-produced SCFAs [73]. These studies together suggest that there exists substantial crosstalk between the host and the microbiome partially mediated by cells of the immune system, particularly on lipid metabolism.

Ultimately, the involvement of the microbiome in macronutrient absorption demonstrates a triad between nutrition, the microbiome, and systemic health. Understanding the role of microbiota-derived metabolites in human health may provide a more precise modulation of human metabolism via microbiome-directed therapies.

## Pharmacological microbiome metabolism

In addition to the previously discussed body of evidence regarding the role of the microbiome in systemic metabolism, we will finally highlight another aspect by which the microbiome strongly impacts host physiology: the metabolism of drugs.

Most drugs are taken orally, and thus will be exposed to the microbiome prior to reaching the blood stream to mediate a therapeutic effect. The microbiome can play a major role in drug metabolism, availability, efficacy, and safety. The microbiome has been shown to alter drugs in myriad mechanisms, including demethylation, deamination, dehydroxylation, deacylation, decarboxylation, oxidation, hydrolysis, deconjugation, and acetylation [74, 75]. The microbiome can also influence drug metabolism indirectly, by impacting the levels of drug-metabolizing enzymes, such as glutathione transferases in the liver and colon [76], or microbial metabolites themselves can compete for the same human enzyme that a drug may be targeting [77]. Since the microbiome has a significant impact on metabolic disease and systemic metabolic homeostasis, this has spurred important studies to elucidate the interaction between the microbiome and the drugs used to treat metabolic disease [78, 79].

### Statins

One group of drugs with major applications in metabolic disease are those that target lipid metabolism in the treatment of elevated triglycerides or cholesterol. Statins (also known as HMG-CoA reductase inhibitors) are a class of cholesterol-lowering agents that reduce mortality in patients at high risk of cardiovascular disease and are the most prescribed drug in the world. Statin therapy is associated with myopathy and T2D in humans, and an initial study in mice found that statin therapy reduced microbiome butyrate production, altered bile acids, and impaired fasting glucose [80]. However, in a large human study, statin therapy negatively correlated with inflammation-associated microbiota Bacteroides2 (Bact2) enterotype [81]; Bact2 dysbiosis is characterized by an increased *Bacteroides:Faecalbacterium* ratio, is associated with inflammatory bowel disease and obesity, and was reduced in obese patients on statin therapy [81]. More work is required to elucidate the interactions and mechanisms by which statin therapy modulates the microbiome.

### Metformin

In T2D, various classes of drugs interact with the microbiome [82]. Metformin is the first-line medication for the treatment of T2D with pleiotropic mechanisms of action. Indeed, more recently it has been suggested that metformin may in part exert a therapeutic benefit by impacting the metabolic activity of the microbiome in patients with type II diabetes [83, 84]. In these studies, it was found that metformin treatment alters the gut microbiome of individuals with treatment-naïve T2D, and the therapeutically beneficial effects of metformin on glucose tolerance could be transferred via fecal transplant from the metformin-treated patients. In mice, metformin was found to decrease *B. fragilis* and increase the bile acid glycoursodeoxycholic acid (GUDCA), and it was suggested that the benefits of metformin were in part mediated by the inhibition of intestinal farnesoid X receptor (FXR) by GUDCA, which was counteracted by *B. fragilis*, and transferable upon fecal transplant from metformin-treated patients [83]. Metformin use also is associated with increased abundance of *Akkermansia muciniphila*, which contributes to better glycemic control [85, 86].

### Other drugs

Given the microbiome’s prime location to interact with orally administered drugs and vast array of metabolic and enzymatic activity, it is notable that interactions have been shown with many medications [79]. In humans, the microbial metabolite p-cresol competes with acetaminophen for sulfonation, a common reaction that many other drugs undergo [77]. High-throughput methodologies show significant promise in uncovering specific interactions between the microbiome and host. Only a small number of microbiome-drug interactions have been elucidated in detail, and recent studies have shown that the range of interactions between the microbiome and xenobiotic compounds may be much larger than previously anticipated [78, 87]. A screen of 1000 marketed non-antibiotic drugs found that 24% of the drugs inhibited the growth of at least one bacterial species of the 40 tested in vitro at physiologically relevant concentrations; all classes of drugs were represented, and antipsychotics were particularly prominent [79]. One path forward is personalized screening for microbiome drug metabolism. Using human fecal cultures combined with HPLC-MS to detect drug metabolism and metabolites, many previous microbially metabolized drugs were confirmed and several new ones identified, including spironolactone, tolcapone, misoprostol, mycophenolate mofetil, capecitabine, hydrocortisone, and vorinostat [88]. In addition to modifying drugs, the microbiome can also modulate the effect of medications by bioaccumulation and sequestration; for example, the antidepressant duloxetine accumulates in several species while also altering metabolite secretion, which in turn affects community composition [89]. The identification and quantification of microbially metabolized drugs offers increased precision and safety moving forward that should be taken into consideration when designing and optimizing therapeutic interventions [90].

These studies offer reason to believe that it is important to evaluate drug exposure in terms of microbiome interactions when developing novel orally active pharmaceuticals in the future. High throughput methodologies represent a paradigm that could be used to scrutinize bioavailability in the context of diverse microbial compositions [91]. These studies could explain some of the less well understood mechanisms of substantially differing responses to commonly administered agents. Perhaps, probiotics could be administered in conjunction with different oral drugs to optimize clinical efficacy.

## Clinical applications, therapeutics, and diagnostics

An important aspect of the identification of microbiome-derived pathways controlling systemic metabolism is that these pathways might be amenable to therapeutic intervention. Microbiome-based therapies are attractive due to their non-invasiveness, low potential for toxicity, and ease of administration. There are a number of promising leads moving forward, whereby discoveries related to the microbiome can be leveraged to hopefully improve human metabolic health in the future.

Currently, the only clinically approved microbiome-based intervention is microbiota transplantation (FMT) in the context of *C. difficile* infection. Community replacement by FMT or antibiotic treatment as therapeutic strategies for obesity and metabolic disease has not proven effective across different studies [92, 93], indicating that more refined approaches are required to modulate systemic metabolism via the gut [94].

Several such refined approaches are actively being investigated, most of them based on live bacteria (probiotics) or their metabolites (postbiotics). A prototypical example for a microbial species-based intervention is the discovery that *Akkermansia* is negatively correlated with obesity [95], which has spurred an effort to test the potential therapeutic benefit of *A. muciniphila* administration. A recent double-blind randomized controlled trial with 32 overweight and insulin-resistant human volunteers provided preliminary evidence to suggest that pasteurized *A. muciniphila* administration may improve insulinemia and plasma cholesterol in overweight humans [96]. *A. muciniphila* can also secrete a protein that induces thermogenesis and GLP-1 secretion in mice fed a high-fat-diet to improve weight and metabolic measures [97]. Similar to preclinical mouse studies, there have also been attempts to transfer a “lean” microbiome into obese individuals; these interventions did not significantly reduce body weight but did improve insulin sensitivity and several other secondary markers [98, 99]. Building off of preclinical observations of microbiota energy harvesting, a recent randomized cross-over dietary intervention and randomized, double-blind, placebo-controlled study found more stool calorie loss in underfeeding vs. overfeeding and with vancomycin treatment vs. placebo [100]. Finally, the effects of bariatric surgery, the most effective treatment for weight loss, were recently found to be partially mediated by the microbial production of a secondary bile acid [101, 102].

These instances of positive evidence notwithstanding, there are a number of challenges that must be overcome related to prebiotic and probiotic interventions, including unresolved mechanisms, conflicting clinical results, and the use of surrogate or subjective study endpoints in clinical trials to date [103]. Regarding mechanism, several explanations have been put forward, including increased SCFAs, hormone signaling, mucus thickness, and barrier integrity along with decreased inflammation, but readouts across different studies have not been consistent. Furthermore, while treatment with single probiotic species is preferable to establish causality [104], some studies using the same mixtures have found conflicting results. For example, the probiotic mixture VSL#3, which contains *Streptococcus thermophilus*, *Lactobacillus*, and *Bifidobacterium* species, has shown metabolic benefits in overweight adults [105] and improved BMI in obese children with non-alcoholic fatty liver disease (NAFLD) [106], while also paradoxically increasing adiposity and weight in obese adolescents [107]. Finally, subjective secondary measures, such as waist circumference, should be avoided. In addition, while some preclinical studies and early-stage clinical trials have been promising, most large-scale human trials of probiotics in weight loss have seen no or marginal benefits [108]. While it is difficult to directly compare probiotic studies due to different probiotics, doses, and duration, several meta-analyses have seen no weight benefits in adults [109, 110] or children [111]. Other meta-analyses have seen statistically significant benefits for overweight adults, but none of the effect sizes observed were larger than 1 kg of weight loss [112–114].

Given the relatively strong preclinical evidence, there are several reasons why probiotic therapies may have had difficulty translating to humans. Mice used in preclinical models are typically young, live in highly controlled environments, equilibrate microbiomes within cages [115], and eat homogenous diets. In contrast, the human microbiome varies dramatically by geography, age, sex, and diet [116, 117]. Variation in the human microbiome determines effective colonization by probiotic species, as some people are highly resistant to engraftment [118].

Given heterogeneous responses to probiotic and other microbiome-targeted therapies, recent studies have suggested that there may be potential in harnessing the microbiome as a diagnostic tool to develop precision medicine treatments for metabolic disorders and obesity. For example, post-prandial glycemic response to diets can by predicted by microbiome composition, opening the possibility of personalized nutrition programs to optimize host metabolism based on microbiota composition [119]. Importantly, recent clinical trials using such personalized interventions demonstrated improved glycemic control and reduced HbA1c in both prediabetic and newly diagnosed T2D when compared to a Mediterranean diet intervention [120, 121]. Understanding how microbial species, gene pathways, and dietary components interact is of upmost importance to harness the power of microbiome data. Two studies that screened the human microbiome for the ability to break down various fiber species identified upregulated gene pathways and species able to outcompete with given nutrients [122, 123]. Individual responses to highly controlled diets containing these fibers in human subjects led to differential changes in the plasma proteome [122]. Similarly, another human trial saw an increase in microbial glycan-degrading genes after a high-fiber diet, while a diet rich in fermented foods increased alpha diversity and decreased markers of inflammation [124]. Two recent studies utilized data from the PREDICT 1 trial with over a thousand patients from the UK, including twin pairs, and a validation cohort in the USA [125, 126]. They found that microbiome composition was a good predictor of postprandial lipid, and to a lesser extent, glycemic response as well as fasting cardiovascular metabolic markers. Varied, plant-based, unprocessed diets were associated with healthier microbiome species, some of the strongest of which were only identified through metagenomic assemblies, emphasizing the need for further characterization. Taken together, these studies suggest that harnessing both taxonomic and metagenomic data to predict individual responses to microbiome-targeting interventions may elucidate and address why intervention responses are so heterogenous and sometimes irreproducible.

In addition to the direct impacts of microbiome composition on host health, we have reviewed numerous examples whereby microbial metabolites influence human health in diverse ways. Modulating these metabolites represents a second therapeutic path to translate microbiome findings. An example for metabolite-based interventions is provided by trimethylamine-N-oxide (TMAO). TMAO is formed from trimethylamine (TMA) produced from microbial metabolism of choline and other TMA-containing molecules. Increased TMAO levels are linked to heart disease, atherosclerosis, T2D, thrombosis, Alzheimer’s disease, and stroke [127–135]. Preclinical targeting of bacterial TMA production was therapeutic in mouse models of atherosclerosis and platelet aggregation [136, 137]. TMAO has been used as a biomarker in several clinical trials [138, 139], and treatments targeting TMAO are now being investigated in chronic kidney disease [140] and cardiovascular disease [141]. TMAO is one of the best-described microbial metabolites, but there are many more that hold therapeutic potential moving forward [142].

Outside of therapeutics, a deeper detailing of the microbiome in patients with various diseases outside of metabolic syndrome could offer valuable information about diagnosis and potential response to standard of care treatments. For instance, the staging and diagnosis of non-alcoholic fatty liver disease has represented a significant challenge in modern medicine. There has been a growing body of evidence which suggests that microbial analysis may lead to minimally invasive approaches to address this challenge. There have been impressive results that have linked dysbiosis to the clinical phenotype of patients with NAFLD-related fibrosis. More specifically, fibrosis has been linked to a decrease in overall microbial diversity and an increase in gram-negative bacteria, which has been postulated to be a source of inflammatory endotoxin [143].

## Conclusions and outlook

The identification of the microbiome impact on systemic metabolism marks the birth of the modern era of microbiome research. In the past 15 years since the discovery that germ-free mice show an abnormal metabolism—including lower blood glucose levels, reduced body fat content, and slower weight gain on high-fat diet compared to conventionally colonized counterparts [4]—the underlying mechanisms have been extensively explored [144]. Possible explanations include the regulation of epithelial lipid uptake by the microbiome [60, 145], the regulation of transcription in metabolic tissues via HDAC3 and mir-181 [39, 146], the regulation of hepatic gluconeogenesis [33], regulation of circadian host biology [147–154], the impact on glucoregulatory viscerofugal enteric neurons [43], and the regulation of insulin signaling [37]. It is likely that all of these mechanisms act in concert and that several other elements of the metabolic microbiota-host crosstalk remain to be discovered.

Systemic metabolism can be understood as the diverse processes that influence physiological energy accounting. The microbiome plays a crucial role as a moderator of these activities. In terms of energy balance, the microbiome not only influences energy harvesting, but also influences neuroendocrine functions, which control hunger and satiety. Of note, the microbiome influence on thermogenesis and energy expenditure has yet to be fully elucidated but represents an important direction for future research. The microbiome has also evolved to interact with each of the three major macronutrient classes in various ways, emphasizing that host metabolite flux cannot be fully interpreted without metagenomic considerations.

Both the promise and the challenge of the field lie in the ability to translate these findings into meaningful clinical interventions, which may take the form of microbiome transplants, pre-, pro-, or post-biotics, metabolite targeting, diagnostics, or precision medicine [155]. In addition to the vast inter-individual and inter-geographical differences in microbiome composition and function in the human population, the lack of a precise understanding of microbiome temporal dynamics and metabolite fluxes between the gastrointestinal lumen and the host systemic circulation presents a formidable challenge. Currently, precision engineering approaches targeting the microbiome largely assume a stable model of host-microbiome interactions, whereby exogenous interventions aimed at modifying microbial community composition will have a durable effect on host metabolites. This is likely not the case. On the basic research side, a detailed understanding of metabolite fluxes between the microbial cells inhabiting the gastrointestinal tract and the eukaryotic cells that compose metabolic tissues would be an important step forward. This can be achieved by the systematic application of metabolite tracing using quantitative flux analysis of labeled molecules. On the clinical side, large-scale multi-center studies, ideally spanning several geographical areas, will be needed to assess the robustness of microbiome-based interventions for metabolic health across different environments, ethnicities, and dietary habits. If the progress of the last 15 years since the initial metabolic characterization of germ-free mice is any indication, the field of metabolic host-microbiome interactions is poised for a decade and a half of deep insights ahead.

## Data Availability

Not applicable.
